# Polyol Pathway Exacerbated Ischemia/Reperfusion-Induced Injury in Steatotic Liver

**DOI:** 10.1155/2014/963629

**Published:** 2014-05-21

**Authors:** Changhe Zhang, Changjun Huang, Yuan Tian, Xiangcheng Li

**Affiliations:** Department of Liver Transplantation, The First Affiliated Hospital of Nanjing Medical University, Nanjing, Jiangsu 210029, China

## Abstract

*Background*. The polyol pathway, a bypass pathway of glucose metabolism initiated by aldose reductase (AR), has been shown to play an important role in mediating tissue ischemia/reperfusion (I/R) impairment recently. Here, we investigated how and why this pathway might affect the fatty liver following I/R. *Methods*. Two opposite models were created: mice with high-fat-diet-induced liver steatosis were treated with aldose reductase inhibition (ARI) and subsequent I/R; and AR-overexpressing L02 hepatocytes were sequentially subjected to steatosis and hypoxia/reoxygenation. We next investigated (a) the hepatic injuries, including liver function, histology, and hepatocytes apoptosis/necrosis; (b) the NAD(P)(H) contents, redox status, and mitochondrial function; and (c) the flux through the caspase-dependent apoptosis pathway. *Results*. AR-inhibition *in vivo* markedly attenuated the I/R-induced liver injuries, maintained the homeostasis of NAD(P)(H) contents and redox status, and suppressed the caspase-dependent apoptosis pathway. Correspondingly, AR overexpression *in vitro* presented the opposite effects. *Conclusion*. The flux through the polyol pathway may render steatotic liver greater vulnerability to I/R. Interventions targeting this pathway might provide a novel adjunctive approach to protect fatty liver from ischemia.

## 1. Introduction

Despite the last hope for selective patients with end-stage liver disease, liver transplantation is facing the dilemma of a huge donor deficit. Today, this worry has been further compounded by the increasing proportion of fatty liver grafts [1]. As fatty livers increase the likelihood of developing postoperation primary nonfunction compared with lean ones, macrovesicular steatosis within more than 30% of hepatocytes is a widely recognized exclusion criterion for candidate liver donors [1, 2]. On the other hand, patients with liver steatosis also have a greater risk of developing liver dysfunction after major hepatectomy, in which portal triad occlusion is routinely utilized to reduce blood loss. The causes of the increased vulnerability of fatty livers in such circumstances have not been fully clarified but may be predominantly associated with excessive oxidative-stress-based injury [1].

The polyol pathway, which consisted of the two key enzymes aldose reductase (AR) and sorbitol dehydrogenase (SDH), has been implicated in the pathogenesis of various diabetic complications. In this bypass pathway, glucose is reduced to sorbitol by AR with the concomitant oxidation of coenzyme NADPH to NADP, and then SDH converts sorbitol to fructose with the reduction of NAD to NADH. Recently, accumulating evidences have indicated the detrimental role of polyol pathway in the I/R events, which could be reversed by ARI [3–5]. Till now, the potential mechanisms underlying this beneficial effect have not been fully elucidated. Meanwhile, liver is rarely involved as the target organ. Therefore, this study was designed to explore how and why the ARI might affect the steatotic liver subjected to ischemia/reperfusion (I/R) insult.

## 2. Material and Methods

The study was approved by the Nanjing Medical University Experimental Animal Department (number NJMU-AEARIA-4001-20120401) and complied closely with the Guide for the Care and Use of Laboratory Animals published by the US National Institutes of Health (NIH publication number 85-23, revised 1996).

### 2.1. Reagents

The AR inhibitor (zopolrestat), oleate, and palmitate were all purchased from Sigma (St. Louis, MO, USA). The triglyceride determination kits were from Sigma Chemical Company. NADP(H), NAD(H), and Annexin V-FITC/PI Quantification Kits were from Biovision. Diagnostic kits for JC-1, malondialdehyde (MDA), glutathione (GSH)/oxidized glutathione (GSSG), reactive oxygen species (ROS), and BCA protein assay kits were all from the Beyotime Institute of Biotechnology (Nanjing, China). Primary antibodies from rabbit directed against Bcl-2, Bax, cleaved caspase 3, and *β*-actin were all purchased from Cell Signaling Technology (Cat. # 3498, 2772, 9664, and 5125, sequentially). The primary antibody from rabbit directed against AR and the secondary antibody from mouse were from Abcam (Cat. # 175394 and 99697, resp.).

### 2.2. Models of Fatty Liver and I/R

Wild-type C57BL/6 male mice (aged 2 weeks and weighted 9-10 g) were obtained from the Chinese Academy of Sciences (SLRC, Shanghai, China), maintained in 25 ± 2°C under a 12 h dark/light cycle, and fed high-fat diet (HFD: 20% lard, 4% sucrose, 2% milk, 1% cholesterol, and 73% standard chow) to develop fatty livers. Six weeks later, the mice were randomized into three groups containing 11 animals each: AR inhibition (ARI) group, pretreated with intraperitoneal administration of zopol (50 mg/kg/day) for 5 days + 70% liver I/R; control (Ctrl) group, treated with saline control + 70% liver I/R; and Sham group, administrated with intraperitoneal administration of zopol (50 mg/kg/day) for 5 days + sham-operation. Before the intraperitoneal injection, one random animal from each group was sacrificed and hepatic sections were stained with Oil Red O to validate the steatosis as previously described [6]. Twenty-four hours after the last drug treatment, the mice were subjected to 1 h of 70% hepatic ischemia and subsequent 6 or 24 h of reperfusion, as described previously [7]. Serum samples were collected to measure alanine aminotransferase (ALT) and aspartate aminotransferase (AST). The liver lobes subjected to I/R were harvested. The animals were ultimately killed under deep anesthesia.

### 2.3. Steatosis Liver Triglyceride Determination

Frozen liver tissues were homogenized and the crude lipids were extracted with the chloroform : methanol (2 : 1). After the removal and blow-drying of chloroform phase, the samples were resuspended in chloroform-Triton X, redried under nitrogen, and eventually resuspended in distilled water. The concentration of triglyceride (TG) was measured in a 96-well plate using a colorimetric assay kit.

### 2.4. Detections of the ROS Generation and Apoptosis/Necrosis of Fatty Liver by Flow Cytometry (FCM)

Single-hepatocyte suspensions were prepared using a modified enzymatic technique as previously described, and subsequent Trypan Blue exclusion demonstrated that cell viability exceeded 89% for all animals [8]. The percentage of ROS-positive hepatocytes was then determined by a FACScan cytometer (Becton Dickinson) using the redox-sensitive dye DCFH-DA, according to the manufacturer's instruction. The degrees of hepatocyte apoptosis and necrosis were also quantified with FCM using the Annexin V-FITC Kit and the Flow J software. The upper right, upper left, and lower right quadrants represented the late apoptotic, necrotic, and early apoptotic cells, respectively. The total proportion of apoptotic cells was then calculated by adding the proportions of late and early apoptosis.

### 2.5. Biochemical Measurements in Fatty Liver

The cytosolic contents of NAD(P)(H) and ratios of NAD/NADH and NADP/NADPH were determined using quantification kits. Fresh liver samples were homogenized in homologous extraction buffer and centrifuged at 14,000 rpm for 5 min. The supernatants were filtered through 10 kD Spin Columns (BioVision, Cat. # 1997-25) to cut off the NAD(P)H-consuming enzyme NADase and then measured according to the manufacturer's instructions.

The concentrations of total glutathione (GSH) and its oxidized disulfide (GSSG) were detected using a second-step enzymatic method, as previously described [9]. The concentration of reduced GSH was obtained by subtracting the GSSG concentration from the total GSH concentration.

To measure MDA, frozen liver samples were homogenized in 20 mM trisbuffer (pH 7.4) containing 5 mM butylated hydroxytoluene and centrifuged at 4000 ×g for 15 min as previously described [10]. All further steps were performed under the guidance of the protocol contained in the commercial kits (Beyotime, Cat. # S0131).

### 2.6. Morphological Observations

Histological sections stained with hematoxylin and eosin (H&E) were processed routinely. The degrees of necrosis and inflammation in the mouse livers were evaluated by one experienced pathologist using the Suzuki score, which measures the extent of congestion, vacuolization, and necrosis on a four-point scale for a total score of 0–12 [11]. For ultrastructural observations, ultrathin (70 nm) slices of the ischemic livers were conventionally prepared for scanning electron microscopy (SEM) and interpreted by a pathologist blinded to the groups.

### 2.7. Model of AR Overexpression, Steatosis, and Hypoxia/Reoxygenation (H/R)

To construct a hepatocellular line stably overexpressing AR, human L02 hepatocytes (Chinese Academy of Sciences, Shanghai, China) were transfected with pcDNA3.1B-AR-Flag-GFP (pAR; Chinese National Human Genome Center, Shanghai, China) using Lipofectamine 2000 (Invitrogen). Before transfection, the following primers were used to amplify the open reading frame of the AR gene: AR-nest-out: 5′-ATTTAAAGGTACGCGCCGCG-3′ (forward) and 5′-CGCTGGCCACTCTACAGGTT-3′ (reverse); AR-nest-in: 5′-ATATCTCGAGATGGCAAGCCGTCTCCTGCT-3′ (forward) and 5′-GCGCGAATTCTAAACTCTTCATGGAAGGGGTAATCC-3′.

To induce the steatosis in L02 hepatocyte, a modified method from previous literatures was deployed [12]. In brief, transfected with pAR (pAR group) or empty plasmid (GFP group), or without transfection (Ctrl group), hepatocytes were incubated in Dulbecco's modified Eagle's medium containing free fatty acids (FFA) at a final concentration of 0.6 mM (2 : 1 ratio of oleate: palmitate) for 8 h to induce fat overloading. Hepatocytes' steatosis was assessed through measuring hepatocellular TG contents using the aforementioned method. Hepatocytes underwent pAR-transfection and no steatosis-induction was used as negative control (Lean group).

The H/R procedure was performed using an AnaeroPack-CO_2_ (Mitsubishi Gas Chemical Co., Tokyo, Japan), as previously described [13]. Firstly, hepatocytes cultured on Petri dish were exposed to phosphate-buffered saline (nutrient deprivation). The dishes were then placed into anaerobic chamber in 37°C environment together with AnaeroPack-CO_2_ which could absorb oxygen and produce carbon dioxide both to an ultimate concentration of 10% within 1 h (oxygen deprivation). Four hours later, the hepatocytes were reexposed to normoxic conditions (95% O_2_ and 5% CO_2_) and total culture medium for a further 6 h.

### 2.8. Investigating L02 Hepatocytes' Apoptosis by FCM Using TUNEL-Staining

To evaluate the hepatocytes' apoptosis after H/R exposure, commercial Apo-BrdU-Red In Situ DNA Fragmentation Assay Kit (BioVision, Cat. # K404-60) was deployed. The Br-dUTP in this kit could actively bind DNA strand breaks, then be identified by a red fluorescence labeled anti-BrdU monoclonal antibody, and ultimately be read by FCM (Ex/Em: 488/576 nm). In brief, L02 hepatocytes after H/R treatment were collected, resuspended into PBS containing 1% (w/v) formaldehyde, and stored in 4°C. Further procedures were performed according to the instructions contained in the kit.

### 2.9. Measuring the L02 Hepatocytes' MDA Level

The apoptosis in L02 hepatocytes was explored using the aforementioned methods when L02 cell underwent H/R treatment.

### 2.10. Mitochondrial Depolarization Assessment

JC-1 probe is a bioimaging dye which dominantly accumulates in the mitochondria as aggregates and emits red fluorescence (580 nm) when mitochondrial membrane potential (ΔΨm) is high. On the contrary, JC-1 flees the mitochondria as a monomer and emits green fluorescence (520 nm) when ΔΨm is low. Here, the mitochondrial depolarization in ischemic liver was observed under a fluorescence microscope using JC-1, as previously described [14]. L02 cells that had not been subjected to H/R were used as the negative control (Sham group).

### 2.11. Immunoblotting Assay

Total proteins from liver tissues subjected to reperfusion for 6 h or H/R-treated L02 hepatocytes were routinely analyzed with immunoblotting. Before sample loading, the BCA Protein Assay Kits were used to assure the homogeneity of protein concentrations. The antibodies were used at the following concentrations: AR (1 : 1500), cleaved caspase 3 (1 : 2000), Bcl-2 (1 : 2000), Bax (1 : 2000), *β*-actin (1 : 2000), and secondary antibody (1 : 2500). The levels of protein expression were determined with Image J software (NIH, Bethesda, MD).

### 2.12. Statistical Analysis

The results are expressed as mean ± SEM. Statistical analysis was performed with an unpaired Student's *t*-test using SPSS 18.0 statistical software. A probability level of *P* < 0.05 was considered statistically significant. All results were obtained from at least 5 independent experiments.

## 3. Results

### 3.1. ARI Protected Fatty Livers against I/R-Induced Impairment and Improved Hepatocyte's Fate

**Figure 1:**
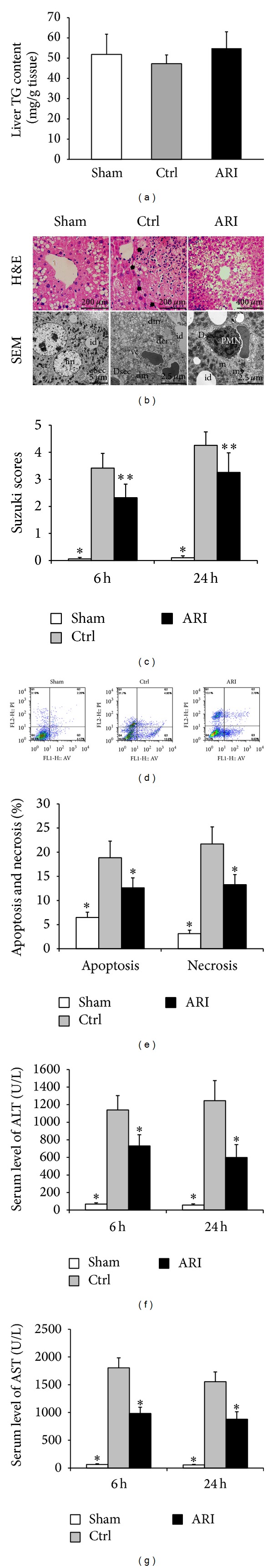
ARI normalized fatty mouse liver function and histology. (a) The hepatic TG levels in Sham, Ctrl, and ARI groups showed no differences after HFD treatment. (b) Both in H&E staining and SEM, ARI group demonstrated less hepatic necrosis, infiltration of inflammatory cells (black arrow), and ultrastructural disturbances relative to the Ctrl group, whereas Sham group showed an almost intact histological architecture; additionally, intrahepatic lipid droplets are clearly visible. (c) In Suzuki score representing the extent of hepatic necrosis and inflammation, Ctrl group was notably higher than Sham group, and this increase was markedly mitigated by ARI administration. (d)-(e) In the flow cytometry, the Ctrl group showed markedly higher proportions of apoptotic and necrotic hepatocytes than Sham group, and ARI significantly reverted these trends. (f)-(g) I/R insult dramatically elevated serum transaminases (ALT and AST) level, and ARI clearly reversed these changes (for each condition, data are expressed as mean ± SEM and analyzed by unpaired Student's *t*-test; *n* = 5, *P < 0.01; **P < 0.05). ld, lipid droplet; hn, hepatocyte nucleus; bc, bile canaliculus; ms, microvillus; sec (dsec), (damaged) sinusoidal endothelial cell; m (dm), (damaged) mitochondria; pmn, polymorphonucleocyte; rbc, red blood cell; der, dilated endoplasmic reticulum; ly, lysosome; vc, vacuole.

To confirm the homogeneity of steatosis in the mouse livers after HFD feeding, their TG contents were analyzed. As shown in Figure 1(a), the TG levels did not differ in the mouse livers among the Sham, Ctrl, and ARI groups. Morphologically, extensive intrahepatic lipid droplets could be observed both in the H&E-stained sections and SEM photography (Figure 1(b)).

As direct causes for liver dysfunction following I/R, hepatocellular necrosis, apoptosis, and inflammation were analyzed. As shown in Figures 1(b) and 1(c), ARI markedly reduced I/R-mediated hepatic necrosis and inflammatory cell infiltration both in morphological observations and in the quantitative Suzuki scores using H&E-stained sections. Analogous results were also observed in flow cytometry deployed to quantitate the proportions of apoptotic and necrotic hepatocytes (Figures 1(d) and 1(e)). Furthermore, the ultrastructural assessment indicated that ARI significantly rehabilitated the I/R-induced histological disruptions (Figure 1(b)).

The transaminases ALT and AST are generated within hepatocytes and drastic elevations always denote cellular membranous leakage or hepatocyte disruption caused by hepatic inflammation and/or necrosis. In the present study, I/R-insult dramatically increased serum transaminase levels, whereas the ARI administration significantly palliated these changes (Figures 1(f) and 1(g)).

**Figure 2:**
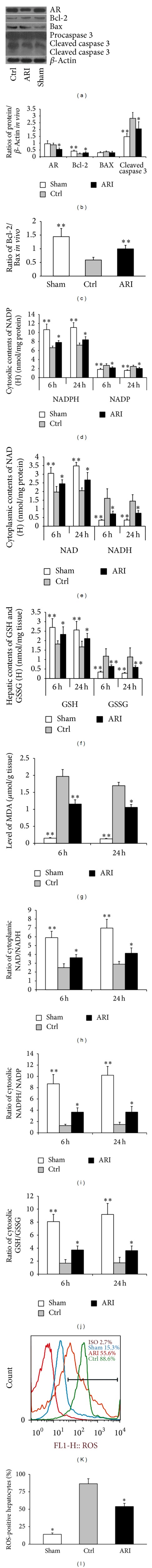
ARI inhibited the caspase-3-dependent apoptosis and reversed the hepatic NAD(P)(H) contents and redox status imbalance while it lowered ROS content. (a) Representative immunostained photograph. (b)-(c) At the protein level, ARI markedly enhanced Bcl-2 and the Bcl-2/Bax ratio while it inhibited cleaved caspase 3 as compared with the Ctrl group, although there was no marked effect on Bax. (d)–(g) After ARI treatment, the I/R-induced decreases in cytoplasmic NAD and cytosolic NADPH and GSH were significantly attenuated, while cytoplasmic NADH and cytosolic NADP and MDA presented the opposite trends. (h)–(j) Remarkable increases could also be observed in the rates of NAD/NADH, NADPH/NADP, and GSH/GSSG after ARI administration. (k)-(l) In flow cytometry used to detect the proportion of ROS-positive hepatocyte, Ctrl group was significantly higher than Sham group, whereas ARI administration markedly attenuated this variation (for each condition, data are expressed as mean ± SEM and analyzed by unpaired Student's *t*-test; *n* = 5, *P < 0.05; **P < 0.01).

It is well known that the proper proportions of Bcl-2 family members, especially the antiapoptotic protein Bcl-2 and the proapoptotic protein Bax, are critical for the maintenance of mitochondrial function and the modulation of the caspase-dependent apoptotic pathway. Meanwhile, caspase 3 is widely accepted as an “executor” for cell apoptosis when it was matured to cleaved form. In this study, ARI markedly enhanced Bcl-2 and the Bcl-2/Bax ratio while it suppressed the activation of caspase 3 at the protein level, although there was no evident change in the Bax protein levels (Figures 2(a)–2(c)).

### 3.2. ARI Reversed the I/R-Mediated Imbalances in NAD(P)(H) and Redox Status

NADPH is an indispensable coenzyme in the generation of GSH, and the latter acts as the major intracellular ROS-scavenger and may in turn inhibit the formation of MDA, a production of membrane lipid-peroxidation. Therefore, the hepatocellular contents of NAD(P)(H), GSH, GSSG, and MDA as well as the proportion of ROS-positive hepatocytes were measured. After ARI treatment, the I/R-induced decreases in the cytosolic content of NADPH and GSH as well as cytoplasmic NAD were significantly attenuated as compared with the control group, while cytoplasmic NADH and cytosolic NADP and MDA contents presented the opposite trends (Figures 2(d)–2(g)). On the other hand, remarkable increases could be observed in the NAD/NADH, NADPH/NADP, and GSH/GSSG rates after ARI administration (Figures 2(h)–2(j)), while the hepatocellular ROS-positive rate presented contrary variation (Figures 2(k) and 2(l)).

### 3.3. AR Overexpression Worsened L02 Hepatocytes' Apoptosis and Elevated Intracellular MDA Level

**Figure 3:**
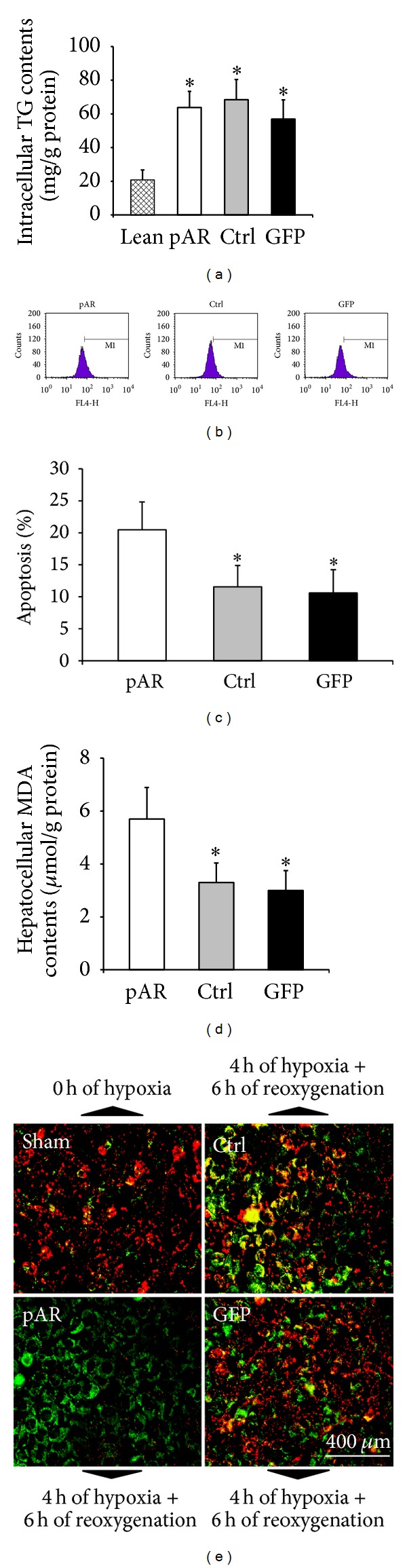
AR-overexpression worsened L02 hepatocytes' apoptosis, elevated intracellular MDA level, and disrupted hepatocytes' mitochondrial membrane potential. (a) After steatosis induction, L02 cell from pAR, Ctrl, and GFP group showed significantly high TG level as compared with Lean group, although there was no marked variance among pAR, Ctrl, and GFP groups. (b)-(c) On FCM using TUNEL staining, AR overexpression evidently deteriorated the H/R-induced apoptosis as compared with Ctrl and GFP group. (d) Meanwhile, pAR transfection also notably elevated the MDA contents when compared to Ctrl and GFP group. (e) In fluorescence using JC-1 staining to detect ΔΨm, the levels of Ctrl and GFP groups were approximately identical, which were clearly lower and higher than those of Sham (Red-fluorescent and cytoplasmic dominance) and pAR group (Green-fluorescent and cytomembrane dominance), respectively (for each condition, data are expressed as mean ± SEM and analyzed by unpaired Student's *t*-test; *n* = 5, *P < 0.01).

After steatosis induction, L02 cell from pAR, Ctrl, and GFP groups showed significantly high TG level as compared with Lean group, although there was no marked variance among pAR, Ctrl, and GFP groups (Figure 3(a)). On FCM using TUNEL staining, AR overexpression evidently deteriorated the H/R-induced apoptosis as compared with Ctrl and GFP groups (Figures 3(b) and 3(c)). Meanwhile, pAR transfection also notably elevated the MDA contents in H/R-treated L02 cell when compared to Ctrl and GFP groups (Figure 3(d)).

### 3.4. AR Overexpression Disrupted the Mitochondrial Membrane Potential

After H/R treatment and JC-1 incubation, Ctrl and GFP groups presented with similar ΔΨm under the fluorescence microscope, being markedly lower than Sham (Red-fluorescent and cytoplasmic dominance) and higher than pAR (Green-fluorescent and cytomembrane dominance) groups (Figure 3(e)).

### 3.5. AR Overexpression Activated the I/R-Mediated Caspase-Dependent Apoptotic Pathway

**Figure 4:**
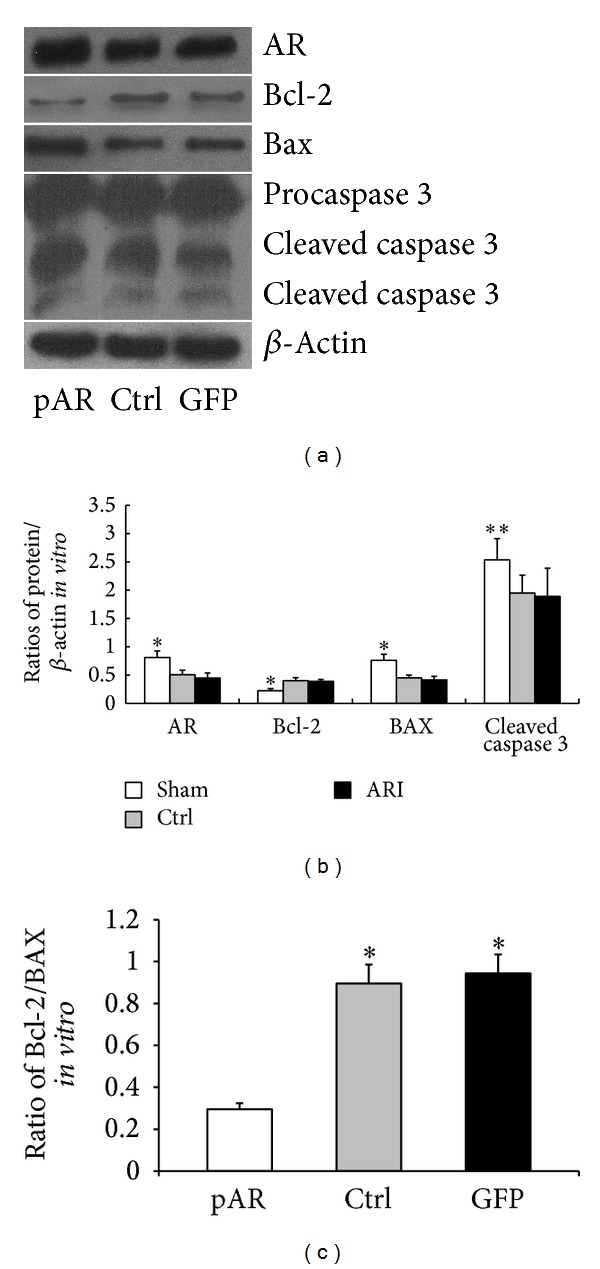
AR-overexpression promoted the activation of caspase 3 in steatosis hepatocytes after H/R. (a)–(c) In western blot, Bcl-2 and Bcl-2/Bax ratio were markedly suppressed by AR overexpressing; whereas Bax and cleaved caspase 3 were enhanced (for each condition, data are expressed as mean ± SEM and analyzed by unpaired Student's *t*-test; *n* = 5, *P < 0.01; **P < 0.05).

As shown in Figures 4(a)–4(c), the Ctrl group and GFP group showed nearly identical Bcl-2 levels and Bcl-2/Bax ratios, which were both higher than those of the pAR group. In contrast, protein Bax and cleaved caspase 3 were significantly higher in the pAR group.

## 4. Discussion

In this study, two opposite models including* in vivo* AR inhibition and* in vitro* AR overexpression were deployed to investigate the role of polyol pathway in ischemic fatty liver. Our results demonstrated that flux through polyol pathway may render steatosis liver greater vulnerability to I/R events, which mainly showed as decreased liver function, disrupted histological architecture, and declined hepatocytes' fates. To be a potential therapeutic target, the role of AR in ischemic steatotic liver should be sufficiently understood and validated.

### 4.1. Improved NADPH/NADP Ratio and Redox Status

NADPH is an indispensable cofactor in the reducing two important antioxidant agents, GSH and thioredoxin, from their oxidized forms. As a result, NADPH acts as an indirect scavenger of ROS in the body [15], and also plays an important role in counteracting the inactivation of the antioxidant enzyme catalase. Therefore, it is reasonable to regard NADPH as an ultimate reducing equivalent and a vital constituent of the mammalian antioxidant defenses [16, 17]. In the present study, the flux through the polyol pathway dramatically reduced the intracellular contents of NADPH and GSH, which in turn worsened the I/R-induced ROS formation, cellular organellar insult (e.g., membrane lipid peroxidation) and mitochondrial dysfunction. Paradoxically, NADPH also contributes to the generation of superoxide under the catalytic effect of NADPH oxidases (NOXs) [17–19]. For this reason, NOXs are generally recognized as the second main source of ROS, only behind the mitochondria. Notably, this phenomenon is almost based on the study of NOXs, but not NADPH [17, 19]. To the best of our knowledge, NADPH itself remains beneficial during I/R because of its robust reducing properties.

### 4.2. Elevated NAD/NADH Ratio

During hypoxia, low level of ATP could occur in the liver to sustain the hepatocytes through anaerobic glycolysis mediated by cofactor NAD [20, 21]. It is plausible that any cause of NAD deficit might exacerbate I/R-induced injury because of the reduced glycolysis. This notion has been supported by several studies, in which NAD depletion following niacin deficiency or poly (ADP-ribose) polymerase overactivation aggravated tissue I/R injury, whereas the subsequent repletion of NAD or niacin markedly reversed this phenomenon [4, 22, 23]. The ameliorative effects of the restored NAD/NADH ratio have also been associated with the suppression of the HIF1*α*-TfR-Tf pathway and the enhanced activation of SIRT1 [5, 14, 24, 25]. Until now, little attention has been given to the NAD/NADH ratio during hepatic I/R, especially in the fatty liver. Limited to the scale of this study, we did not explore all the courses discussed above, but the thread of NAD began to loom. The flux through the polyol pathway worsened I/R-induced NAD depletion in the fatty liver, which may impair NAD-dependent glycolysis and aggravate energetic failure. Additionally, fatty livers intrinsically have a lower NAD/NADH ratio than lean livers, which might account in part for their increased vulnerability to I/R-insult [26].

### 4.3. Cross-Talk between Redox Imbalance, Mitochondrial Impairment, and Hepatocyte Fate

Mitochondrion acts as the fundamental generator of intracellular energy and is also involved in multiple hepatocellular function regulations such as redox homeostasis and cell fate [27]. On the other hand, hepatocyte necrosis and apoptosis represent two major direct causes of liver dysfunction following severe I/R impairment and represent different extremes on a continuum of cell death. They share the common triggers of mitochondrial impairment and resultant energy failure [28]. During this cascade, excessive ROS and lipid peroxidation, together with an imbalance in pro- and antiapoptosis proteins (e.g., Bax and Bcl-2, respectively), promote the mitochondrial permeability transition [27, 29]. These changes may, in turn, initiate and interact with the following mitochondrial events: respiratory chain uncoupling, the release of cytochrome *c*, the downstream activation of caspase 3, and so forth [27]. In the present study, the increased Bax/Bcl-2 ratio which resulted from AR overexpression clearly lowered the mitochondrial membrane potential and contributed to the overactivation of the caspase pathway. However, ARI treatment significantly alleviated the I/R-induced increase of ROS, Bax/ Bcl-2 ratio, and activation of caspase 3, which could eventually improve the liver function. Interestingly, besides its bioactivity in converting glucose to sorbitol, the polyol pathway can also detoxify the noxious aldehydes generated by lipid peroxidation [30]. Therefore, it seemed reasonable that the polyol pathway may protect tissues from I/R-injury by downregulating the intracellular aldehyde content. This notion has been supported by I/R model in which ARI increased the cytosolic aldehyde content and exacerbated tissue ischemic injury [30]. Presumably, these discrepancies may be attributed to the differences in tissue resources, inhibition schemes, the duration of ischemia or reperfusion, and so forth. Besides the polyol pathway, there are many other enzymes responsible for the detoxification of aldehydes, after all [31, 32].

Until now, various protocols have been developed to modulate the metabolic processes and/or antioxidant properties of the fatty liver during I/R. As the mitochondrion acts as both major victim and the generator of oxidative stress, pharmacological agents aiming at keeping the hepatic energetic balance and mitochondrial function may represent a novel strategy for protecting the fatty liver from severe I/R-induced impairment.

## 5. Conclusion

In conclusion, we used two opposite models,* in vivo* AR inhibition and* in vitro* AR overexpression, to investigate the effects of AR on the fatty liver after I/R. Our study demonstrated that the flux through the polyol pathway renders the fatty liver greater vulnerability to I/R in the forms of decreased liver function and deteriorated hepatocytes fates, which could be evidently rehabilitated by ARI pretreatment. This study demonstrated that strategies that inhibit polyol pathway might provide a novel adjunctive approach to protect the steatotic liver from I/R-insult.
